# Galectin network in osteoarthritis: galectin-4 programs a pathogenic signature of gene and effector expression in human chondrocytes in vitro

**DOI:** 10.1007/s00418-021-02053-1

**Published:** 2021-11-30

**Authors:** Katharina M. Pichler, Anita Fischer, Jürgen Alphonsus, Catharina Chiari, Sebastian Schmidt, Michael Kenn, Wolfgang Schreiner, Daniela Weinmann, Mario Rothbauer, Reinhard Windhager, Hans‑Joachim Gabius, Stefan Toegel

**Affiliations:** 1 Karl Chiari Lab for Orthopaedic Biology, Department of Orthopedics and Trauma Surgery, Medical University of Vienna, Waehringer Guertel 18-20, 1090 Vienna, Austria; 2 Department of Orthopedics and Trauma Surgery, Division of Orthopedics, Medical University of Vienna, 1090 Vienna, Austria; 3 Center for Medical Statistics, Informatics and Intelligent Systems, Institute of Biosimulation and Bioinformatics, Medical University of Vienna, Vienna, Austria; 4 Institute of Physiological Chemistry, Faculty of Veterinary Medicine, Ludwig-Maximilians-University Munich, 80539 Munich, Germany; 5 Ludwig Boltzmann Institute for Arthritis and Rehabilitation, Vienna, Austria

**Keywords:** Inflammation, Lectin, NF-κB, Cartilage, 3D spheres, Primary cells

## Abstract

Galectin-4 (Gal-4) is a member of the galectin family, which have been identified as galactose-binding proteins. Gal-4 possesses two tandem repeat carbohydrate recognition domains and acts as a cross-linking bridge in sulfatide-dependent glycoprotein routing. We herein document its upregulation in osteoarthritis (OA) in correlation with the extent of cartilage degradation in vivo. Primary human OA chondrocytes in vitro respond to carbohydrate-inhibitable Gal-4 binding with the upregulation of pro-degradative/-inflammatory proteins such as interleukin-1β (IL-1β) and matrix metalloproteinase-13 (MMP-13), as documented by RT-qPCR-based mRNA profiling and transcriptome data processing. Activation of p65 by phosphorylation of Ser536 within the NF-κB pathway and the effect of three p65 inhibitors on Gal-4 activity support downstream involvement of such signaling. In 3D (pellet) cultures, Gal-4 presence causes morphological and biochemical signs of degradation. Taken together, our findings strongly support the concept of galectins acting as a network in OA pathogenesis and suggest that blocking their activity in disease progression may become clinically relevant in the future.

**Supplementary Information:**

The online version contains supplementary material available at 10.1007/s00418-021-02053-1.

## Introduction

Efforts to identify pathogenic mechanisms of diseases are increasingly guided by the concept of the sugar code. Ubiquitous presence, structural complexity, and fine-tuned regulation of expression among the glycans of cellular glycoconjugates provide good reason to consider them as molecular messages (Gabius (ed.) 2009; Gabius and Roth 2017). The glycan-encoded information is “read” by endogenous receptors (lectins), and the generated glycoconjugate-lectin complexes then elicit various post-binding effects such as outside-in signaling toward context-dependent outcomes (Lis and Sharon 1998; Kaltner et al. 2019). To exploit the structural diversity of glycans, the duplication and diversification of ancestral genes for lectins during phylogenesis has led to diverse families. Their members differ in the sequence of the carbohydrate recognition domain (CRD). In aspects of modular architecture, as seen for example in C-type lectins (Weis et al. 1998; Gready and Zelensky 2009; Mayer et al. 2017), structural and functional studies of each protein are necessary. Looking at lectins in molecular medicine, a key question is whether their expression profiles are separate or coregulated, in the latter case prompting the assumption for functional cooperation.

When focusing on the adhesion/growth-regulatory galectins, vertebrates present the common β-sandwich-type CRD in three types of modular arrangements. The current status of mapping their expression already documents overlaps, making it likely that more than one galectin is present locally (Cooper 2002; Kaltner et al. 2017; Hirabayashi (ed.) 2018). Consequently, investigations on the involvement of galectins in (patho)physiological processes should include respective expression profiling at an early stage of research, as this would provide a clear direction for ensuing systematic and functional analyses of each detected galectin (Habermann et al. 2021). The interest to do so for galectins in osteoarthritis (OA), a disease of urgent public health concern, is explained by two sets of observations: (i) the suggested role of certain galectins in chicken chondrogenesis and chondrocyte hypertrophy as well as in chondrocyte survival in mice (Matsutani and Yamagata 1982; Nurminskaya and Linsenmayer 1996; Colnot et al. 1999, 2001; Bhat et al. 2011), and (ii) the presence of galectin-3 (Gal-3) on chondrocyte surfaces and its association with disease manifestation in animals (Reboul et al. 2004; Janelle-Montcalm et al. 2007; Boileau et al. 2008). This body of solid evidence led us to immunohistochemical galectin fingerprinting of human OA knee cartilage, which revealed expression of Gal-1 and Gal-3, less so for Gal-4 and Gal-8, with cell positivities found to be significantly different between regions of low and high degrees of degradation (Toegel et al. 2014). This work on patient material suggested an involvement of the detected galectins in OA pathogenesis. Of note, ensuing studies revealed that galectins are capable of stimulating matrix metalloproteinases (MMPs) including MMP-13, which is very efficient in degrading type II collagen and which is specifically expressed in the cartilage of human OA patients, thus contributing to worse outcomes in experimental (Wang et al. 2013) and clinical OA (Hu and Ecker 2021). Having disclosed, in stepwise fashion, the activity of Gal-1, Gal-3, and Gal-8 as inducers of pro-degradative/-inflammatory effectors in OA chondrocytes (Toegel et al. 2016; Weinmann et al. 2016, 2018; Pichler et al. 2021), the question on the functional status of Gal-4—which was revealed to be expressed at comparable levels as Gal-8 in OA chondrocytes (Toegel et al. 2014)—remains to be answered.

Gal-4 is a heterobivalent protein first described in swine oral epithelium (Chiu et al. 1992, 1994) and rat intestine (Oda et al. 1993). Its strong affinity to sulfatide and complex-type N-glycan termini with unsubstituted N-acetyllactosamine has been attributed to its role in routing and delivery of glycoproteins in apical and axonal transport (Ideo et al. 2005; Delacour et al. 2005; Velasco et al. 2013; Murphy et al. 2021). In colon cancer cells, Gal-4 has been defined as a suppressor, for example, inducing cell cycle arrest and reducing chemokine/cytokine presence (Satelli et al. 2011; Michalak et al. 2016, 2019; Rao and Rao 2017). In contrast, the switch from branched core 2 to Gal-4-binding core 1 mucin-type O-glycans, to which the 3′-*O*-sulfated disaccharide suTF antigen (CD176su) belongs, has been reported to drive metastatic castration-resistant prostate cancer (Tzeng et al. 2018) and to exacerbate intestinal inflammation induced by Gal-4 (but not Gal-3) in CD4^+^ intestinal T cells by stimulating interleukin-6 (IL-6) production (Hokama et al. 2004; Nishida et al. 2012). On activated human neutrophils, Gal-4 was found to be bioactive in triggering phosphatidylserine exposure as Gal-1 and Gal-2 do, albeit through different signaling (Stowell et al. 2007). Overall, these results indicate cell-type specificity of Gal-4-elicited responses. They preclude making reliable predictions for this galectin’s role in OA. The present study was designed to close this gap.

Thus, we have first extended expression monitoring of Gal-4 in OA patients to strengthen the case for a correlation of the level of its presence with the disease status in vivo, and then examined the hypothesis of a trigger capacity of Gal-4 in OA chondrocytes in vitro. Profiling gene expression and production of disease markers as well as measuring the size of 3D (pellet) cultures, a sensitive indicator of pathogenic activity (Pichler et al. 2021), identified a pro-degradative/-inflammatory capacity of Gal-4 that involves downstream signaling via the NF-κB route.

## Materials and methods

*Galectins.* Human Gal-1, Gal-3, Gal-4, and Gal-8 proteins were obtained by recombinant production and purified by affinity chromatography of lactose-presenting resin as a crucial step, using one- and two-dimensional gel electrophoresis and gel filtration to ascertain purity (André et al. 2007; Sarter et al. 2009). Labeling by commercial fluorescent dyes was performed under activity-preserving conditions as described (Kaltner et al. 2015). The anti-Gal-4 antibody preparation was checked by systematic enzyme-linked immunosorbent assays (ELISAs) for cross-reactivity against other human galectins, and cross-reactive material was removed chromatographically using protein-loaded beads (Langbein et al. 2007).

*Clinical specimens and cell culture.* Human articular cartilage specimens were obtained from OA patients during total knee replacement surgery with written informed consent and following the terms of the ethics committee of the Medical University of Vienna (EK-No. 1822/2017 and 1555/2019). Chondrocytes were isolated from femoral condyles and tibial plateaus and cultured in growth medium [DMEM GlutaMAX (Gibco) supplemented with 10% fetal calf serum (Biochrom), 1% penicillin/streptomycin (Gibco), and 0.1% amphotericin B (Sigma)] in a humidified atmosphere of 5% CO_2_/95% air at 37 °C. For all assays, primary chondrocytes were used without subculturing to preserve the chondrocyte phenotype. For monolayer experiments, chondrocytes at 90% confluence were serum-starved overnight and then treated with galectins for 24 h. NF-κB pathway components were inhibited using Bay 11–7082 (Merck), IKK inhibitor VII (Merck), and CAPE (Merck). Concentrations of reagents and time periods of treatments were used as indicated in the figures or figure legends. 3D pellet formation was previously described in Pichler et al. (2021). In brief, 5 × 10^5^ chondrocytes per pellet were used and cultured in a growth medium. After two days, they were transferred into a starvation medium [DMEM GlutaMAX, 1% penicillin/streptomycin mixture, 0.1% amphotericin B and 1% insulin-transferrin-selenium (Gibco)]. After three weeks of culture in starvation medium, pellets were treated with Gal-4 (12 µg/ml) for two weeks. The pellet size was measured with a Nikon ECLIPSE TE2000-U microscope (×2 magnification) and NIS-Elements software at treatment start and end. In addition, culture supernatants were collected at the end of treatment to determine the secretion profile of marker proteins by Luminex assay, and mRNA of pellets was isolated to perform RT-qPCR.

*Histology and immunohistochemistry.* Cartilage tissue preparation, assessment of degeneration, and immunohistochemical galectin staining were previously described (Mankin et al. 1971; Toegel et al. 2014). In brief, deparaffinized cartilage tissue sections were stained with Safranin O and graded according to the Mankin score (MS). Consecutive sections were assessed by two independent observers for the percentages of Gal-4-positive chondrocytes in the regions of interest after immunohistochemical staining. Therefore, tissue sections were incubated with rabbit polyclonal antibodies against human Gal-4. The staining was developed using horseradish peroxidase-containing reagent (VECTASTAIN Elite ABC Kit, Vector Labs) together with 3,3′-diaminobenzidine tetrahydrochloride hydrate (Fluka) and H_2_O_2_ as substrates. Sections were counterstained using Mayer’s hemalum solution (Merck). The sections were observed on a Zeiss Axio Imager M2 microscope equipped with Zeiss EC Plan-Neofluar 20×/0.5 and N-Achroplan 5×/0.15 objectives, and digital images were captured using a Zeiss Axiocam 506 color camera (2752 × 2208 pixels; pixel size 4.54 × 4.54 µm). White balance was adjusted automatically using the ZEN software. No additional editing was performed.

*Fluorescence cell staining.* Chondrocytes grown as monolayer cultures were trypsinized to obtain a cell suspension of 3 × 10^5^ cells in 50 µl phosphate-buffered saline (PBS). Cells were incubated at 4 °C for 10 min with 5 µg/50 µl AlexaFluor488-labeled Gal-4 in the presence or absence of 0.1 M lactose. Images were immediately taken without fixation using laser scanning microscopy (Zeiss, LSM700). Images were collected using a 63×/1.4 Plan-Apochromat, Oil DIC M27 objective lens with a pinhole of 1 Airy unit. The following image acquisition conditions for AlexaFluor-488 staining were used with the aid of the ZEN 2012 black software: detector gain, 900; bit data depth, 12; scan averaging number, 4; scan speed, 6.

*RT-qPCR.* Isolation of total RNA, cDNA synthesis, and SYBR-green-based qPCR experiments was performed as described previously (Toegel et al. 2013, 2014). Briefly, total RNA was isolated using the innuPREP RNA Mini Kit. Each RNA sample was examined for purity and quantity using the NanoDrop 2000 system before reverse transcription into cDNA. The protocols followed the minimal guidelines for the design and documentation of RT-qPCR experiments (Bustin et al. 2010). A detailed checklist containing all relevant information is provided by the authors upon request. mRNA levels were calculated as relative quantities compared to the untreated controls considering amplification efficiencies and normalization to succinate dehydrogenase complex, subunit A (SDHA), which had been identified as a stable reference gene under the experimental conditions of this study.

*Luminex*. For secretion analysis of IL-6, IL-8, MMP-1, MMP-3, and MMP-13, a 5-plex human Magnetic Luminex Assay (LXSAHM-05, R&D Systems) was used. Cell culture supernatants were diluted 1:2 in Calibrator Diluent RD6-52 after centrifugation at 16000×*g* for 4 min and analyzed according to the manufacturer's standard preparation protocol using a Luminex™ MAGPIX™ System (Invitrogen). Target protein concentrations were calculated from the respective target protein's standard curves using GraphPad Prism 7 (GraphPad Software Inc.) with a third-order polynomial cubic interpolation function.

*Computational profiling of promoter and intron regions for regulatory elements.* The proximal promoter (2500 bp) and nine introns of the human Gal-4 gene were examined for the presence of sequence motifs with a putative affinity for transcription factors by the MatInspector software (Matrix Library 10.0) with stringent settings to ensure high-quality scores, as previously reported for Gal-1, Gal-3, and Gal-8 to ensure comparability (Toegel et al. 2016; Weinmann et al. 2016, 2018).

*Microarray and bioinformatics analyses.* Human OA chondrocytes were isolated from five female patients (62–68 years). Cells were cultured in 25 cm^2^ flasks. Following overnight serum starvation, cells were incubated with 100 µg/ml Gal-4 for 24 h. Control cells were not treated. RNA quality and quantity were controlled (A260/A280: 1.98–2.07; RNA integrity numbers: 9.6–10). The microarray data discussed in this publication are deposited in the GEO database with the number GSE183531. Robust multi-array average (RMA) normalization was performed by the affy package (Gautier et al. 2004) from Bioconductor. By the limma package (Ritchie et al. 2015) a sorted list of probe sets was obtained. After mapping probe sets to Entrez Gene IDs, they were sorted by descending significance. To define a respective set of highly regulated genes, the list was truncated for fold change (FC) > 2 and *p* < 0.05. For heatmap illustration, paired samples were evaluated between control and Gal-4 treatment (i.e., samples 1 and 6, samples 2 and 7, and so on). Paired differences were filtered for |log2(FC) > 2|. Gene set enrichment analysis (GSEA) (Subramanian et al. 2005) was performed against the c3.tft.v7.0 database, using permutation type = “gene_set,” and a seed = 149. Pathways regulated with normalized enrichment score (NES) > 2 were retained. In addition, Cytoscape (Shannon et al. 2003; Isserlin et al. 2014) was used to display pathways regulated by Gal-4 with NES > 2. Node size reflects the number of genes in the corresponding pathway (# nodes).

*In-Cell Western (ICW) assay.* The ICW assay was performed using the Odyssey Imaging System (LI-COR Biosciences) according to a previously described protocol (Elshamly et al. 2019). In brief, chondrocytes were grown in 96-well plates, and starved cells underwent Gal-4 (50 µg/ml) treatment for 1 h. Cells were washed and fixed with methanol (–20 °C) for 10 min. Cells were washed with PBS, and sites for nonspecific protein binding were blocked with LI-COR Odyssey blocking buffer for at least 90 min. Primary antibodies were incubated overnight at 4 °C diluted in blocking buffer (NF-κB p65/mouse, Cell Signaling #6956, 1:1000 and p-NF-κB p65 (Ser536)/rabbit, Cell Signaling #3033, 1:800). On the next day, cells were washed and incubated subsequently with solution containing the secondary and staining antibody (donkey anti-mouse IgG IRDye™ 680RD, 1:1000 and goat anti-rabbit IgG IRDye™ 800CW, 1:1000 from LI-COR Biosciences) diluted in blocking buffer containing Tween 20 (0.2%) for 1 h at RT. The plate was dried and scanned with the Odyssey CLx Infrared Imaging System (LI-COR Biosciences). Signals were analyzed using the software provided by the manufacturer (Image Studio Version 5.2, LI-COR Biosciences). p65 phosphorylation was normalized to the total amount of the corresponding protein.

*Statistics.* SPSS 27.0 was used to analyze the correlation between Gal-4 immunopositivity and the MS. Scatterplots of percentage of Gal-4-positive cells versus Mankin scores were prepared for each patient. The Pearson´s correlation coefficient was calculated for each of the 19 patients, and the *t*-test was used to assess whether the distribution of correlation coefficients (normal distribution verified using Shapiro–Wilk test) was significantly different from 0. qPCR data were analyzed using the Shapiro–Wilk test followed by the Wilcoxon signed-rank test or the Friedman test.

## Results

### Gal-4 presence correlates with cartilage degeneration

**Fig. 1:**
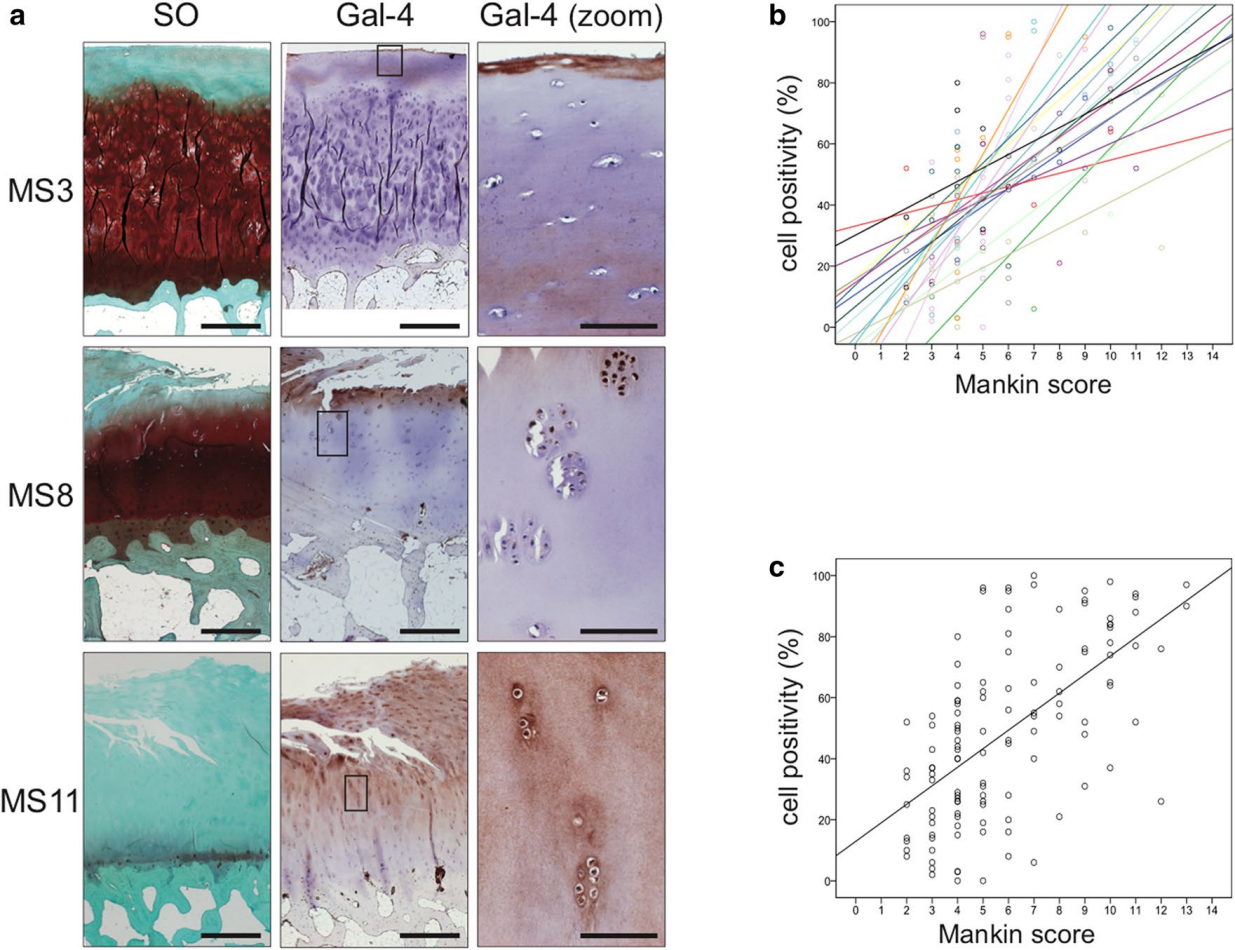
The presence of Gal-4 in chondrocytes correlates with the degeneration of cartilage. **a** Histological sections of articular cartilage were stained with Safranin O. From each patient, several regions of interest were carefully graded according to the Mankin score (MS). Shown are three representative tissue sections of specimens with MS3, MS8, and MS11, respectively. Consecutive counterstained sections were immunohistochemically processed for Gal-4 detection (brown). Scale bars: first and second column: 500 µm, third column: 100 µm. **b, c** A scatterplot of Mankin scores versus the percentages of Gal-4-positive cells in the cartilage of 19 patients, with regression lines for each patient (**b**) or across all patients (**c**), is shown. The determined MS ranged from 2 to 13 and the Gal-4 positivity ranged from 0 to 100%

Specimens of osteoarthritic cartilage from 19 patients were assessed to determine histopathological signs of cartilage degradation. Consecutive tissue sections were processed with anti-Gal-4 antibody, and the percentage of cell positivity was determined (0–100%) after staining. Figure 1a exemplarily illustrates three different stages of cartilage degeneration from mild to severe. MS3 has characteristically almost intact surface and tissue morphology, where articular chondrocytes were hardly positive for Gal-4 from the superficial zone to the deep zone. Regions of MS8 presented considerable surface discontinuity and matrix depletion with mostly positive chondrocytes in the superficial zone. Notably, signal presence for Gal-4 in chondrocytes diminished towards the middle zone. At MS11, chondrocytes appear in clusters, while vertical clefts and matrix loss occur with an intense staining for Gal-4 of all chondrocytes from the superficial zone to the middle zone. Scatterplots relating MS to cell positivity were drawn for each patient (Fig. 1b) and the corresponding Pearson´s correlation coefficients ranged from 0.450 to 0.937 (mean: 0.725 ± 0.163). Positive correlation was delineated for each patient and the entire group (Fig. 1c; *p* < 10^−12^, *t*-test). These results demonstrate that the percentage of Gal-4-positive chondrocytes increased with the severity of cartilage degeneration, drawing attention to examining promoter and intron sequences of the Gal-4 gene as a basis for insights into regulability.

### Monitoring promoter and intron sequences for putative regulatory elements

Computational processing by a respective algorithm (MatInspector) detected a panel of sequence motifs with putative activity for binding transcription factors (Supplementary Material, Table SI). With this data set in hand, a comparison of Gal-4 with respective information for Gal-1, Gal-3, and Gal-8 was possible. Shared motifs are listed in Supplementary Material, Table SII. Considering the pathophysiological relevance of transcription factor expressions in OA, known candidates as regulators and the representation of respective sequence motifs are given in Supplementary Material Table SIII. The large number of the sign ( +) for positivity present in this table among galectin genes supports the concept of their involvement in OA pathogenesis and prompted us to study the impact of Gal-4 binding to OA chondrocytes, starting with documenting its carbohydrate-dependent cell surface association.

### Effects of Gal-4 binding

**Fig. 2:**
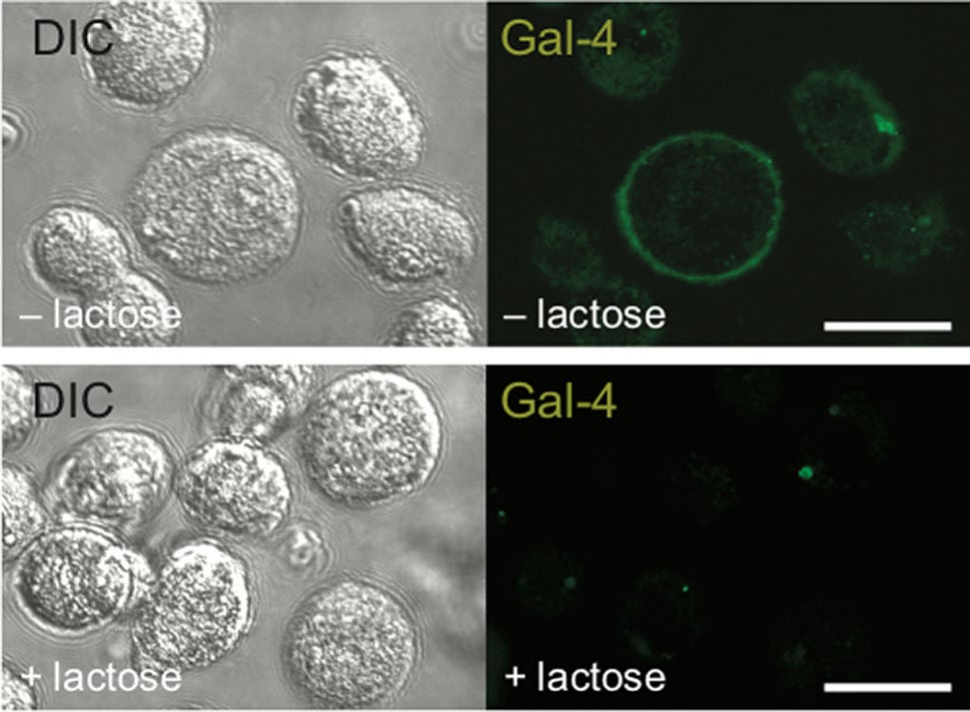
Surface binding of fluorescent Gal-4 to OA chondrocytes in vitro. Cultured chondrocytes were trypsinized and resuspended prior to their treatment with AlexaFluor488-labeled Gal-4 in the presence (upper row) or absence (lower row) of lactose. After washing, the cells were analyzed using a Carl Zeiss LSM 700 laser scanning microscope at ×630 magnification. Representative fluorescence images (right) and corresponding differential interference contrast images (DIC; left) are presented. Scale bars: 20 µm

**Fig. 3:**
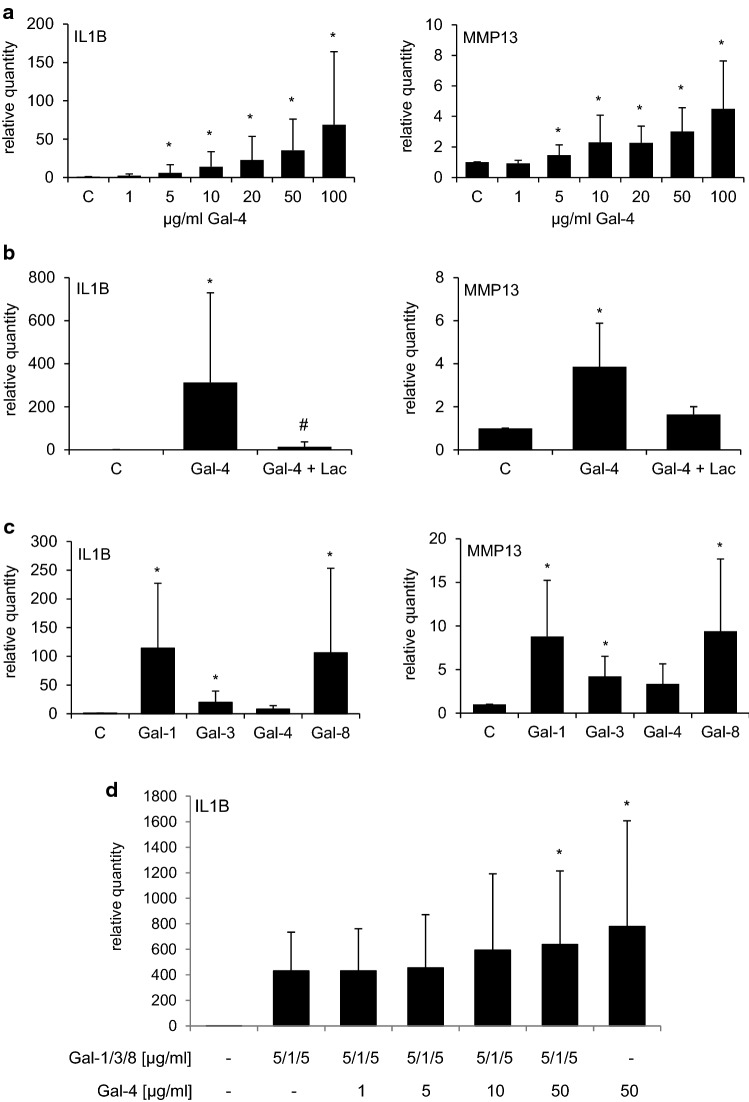
Gal-4 upregulates transcription of genes for pro-inflammatory mediators in OA chondrocytes. **a** OA chondrocytes (*n* = 8 patients) were treated with different concentrations (1, 5, 10, 20, 50, 100 µg/ml) of Gal-4 for 24 h. SDHA was used as reference gene to compute relative quantities of IL1B or MMP13. Asterisks mark significant upregulation after galectin treatments (*p* < 0.05, Wilcoxon test). **b** OA chondrocytes (*n* = 6 patients) were treated for 24 h with 50 µg/ml Gal-4. In parallel, samples were co-treated for 24 h with 0.2 M lactose. SDHA was used as reference gene. Asterisks mark significant differences between control and treatments. Number signs mark significant differences between galectin-treated cells and lactose-galectin-treated cells. (*p* < 0.05, Friedman test). **c** OA chondrocytes (*n* = 5 patients) were treated for 24 h with 10 µg/ml Gal-1, 18 µg/ml Gal-3, 24 µg/ml Gal-4 or 24 µg/ml Gal-8S. SDHA was used as reference gene. Asterisks mark significant differences between the control and galectin treatments (*p* < 0.05, Friedman test). **d** OA chondrocytes (*n* = 4) were treated for 24 h with a mixture of 5 µg/ml Gal-1, 1 µg/ml Gal-3, and 5 µg/ml Gal-8 in presence or absence of increasing concentrations of Gal-4. Asterisks mark significant differences in comparison to Gal-1/-3/-8 treated cells (*p* < 0.05, Wilcoxon test)

OA chondrocytes were visualized using laser scanning microscopy with differential interference contrast (DIC) or the fluorophore channel. OA chondrocytes incubated with fluorescent Gal-4 presented surface staining, as illustrated in Fig. 2 (upper panel). Binding was blocked by the presence of the cognate sugar lactose (lower panel). Cell viability was not impaired by increasing Gal-4 doses, thus excluding toxicity (Supplementary File 1, Fig. S1). At the mRNA level, Gal-4 binding promoted the transcription of IL1B and MMP13 in a dose-dependent and lactose-inhibitable manner (Fig. 3a, b). Further Gal-4-regulated pro-degradative (MMP1, MMP3, and ADAMTS4) and matrix-synthesizing genes (ACAN, COL1A1, and COL2A1) are illustrated in Supplementary File 1, Fig. S2. To directly compare Gal-4 with Gal-1, Gal-3, and Gal-8 activity, we treated OA chondrocytes with equimolar concentrations of these proteins and monitored the levels of IL1B and MMP13 induction (Fig. 3c). Gal-4 caused the mildest changes on the regulation of IL1B and MMP13; however, when OA chondrocytes were treated with a mixture of Gal-1/-3/-8, together with Gal-4, a further increase was detected (Fig. 3d). This additive effect should be highlighted as it indicates an interaction of all four galectins in promoting the pathomechanisms of OA. To investigate the specific role of Gal-4 in more detail, Gal-4-dependent reprogramming of gene expression was studied next by transcriptomic analysis.

### Microarray data reveal pathways stimulated by Gal-4

**Fig. 4:**
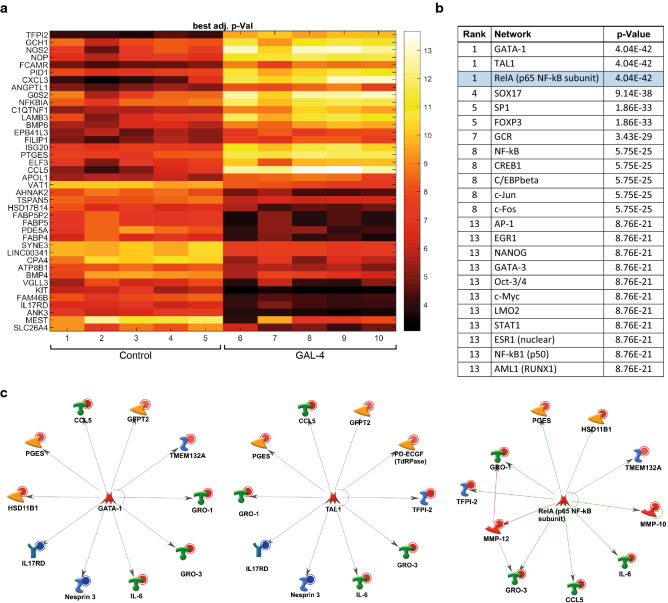
Microarray data reveal Gal-4-regulated genes and networks of five OA patients. **a** Heatmap shows log2-expression values (color scale bar on the right) of the top 20 upregulated and top 20 downregulated genes. **b** The table shows the top 20 networks out of all genes significantly regulated by Gal-4, computed by the transcription regulation algorithm of MetaCore. *P*-values represent probabilities that relations between regulated genes and networks have occurred by chance. Equal ranks were assigned to networks with equal *p*-values. **c** The most important transcription regulation networks from all genes significantly regulated by Gal-4. GATA-1 (left), TAL1 (middle), RelA_p65NFkb (right)

**Fig. 5:**
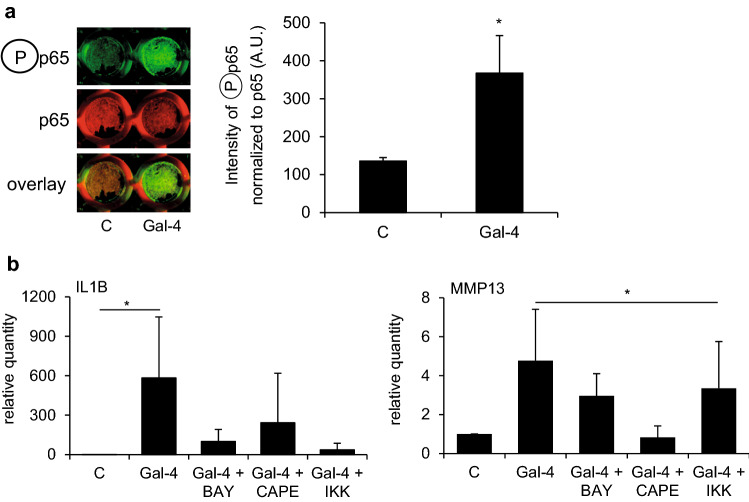
Gal-4 activates the NF-κB pathway in OA chondrocytes. **a** OA chondrocytes from four patients were exposed for 60 min to Gal-4 (50 µg/ml), and NF-κB activation was measured by In-Cell Western (2 technical replicates). A scan of cells from one representative patient is shown. Signal intensities of phosphorylated p65 were normalized to total p65 and shown as mean values and standard deviations (*n* = 4). Significant differences to untreated control cells set to 1 are indicated with asterisks (*p* < 0.05, one-sided paired *t*-test). **b** Bar charts show relative quantities (mean values and standard deviations, *n* = 4) of mRNA levels of IL1B (left) and MMP13 (right), measured using RT-qPCR (2 technical replicates) with untreated control values set to 1. Cells were treated for 24 h with Gal-4 (50 µg/ml) alone or in combination with specific NF-κB inhibitors (4 µM Bay 11–7082, 40 µM CAPE, or 4 µM IKK inhibitor VII). Significant differences between groups are indicated with asterisks (*p* < 0.05, *n* = 4, Friedman test)

The binding of this galectin to OA chondrocytes affected mRNA levels significantly. The top 20 up- and downregulated genes are illustrated as a heatmap in Fig. 4a. Using the transcription regulation algorithm of MetaCore software, GATA-1, TAL1, and RelA_p65NFkb were delineated as possibly involved in regulation (Fig. 4b). Notably, all three networks share rank 1 (for corresponding network maps, see Fig. 4c). In line with this, an enrichment map implicated NF-κB signaling as a downstream response to Gal-4 binding (Supplementary File 1, Fig. S3). Next, all genes significantly regulated by Gal-4 were subjected to consideration in MetaCore’s “canonical pathway maps,” “process networks,” and “diseases,” revealing immune responses and inflammation as common hits (Supplementary Material, Tables SIV–SVI). To experimentally validate the assumption of NF-κB involvement, we tested the hypothesis of this pathway’s activation. Site-specific phosphorylation of p65 at Ser536 after Gal-4 treatment was revealed by In-Cell Western, and the signal intensity was quantified compared to the signal of total p65 (Fig. 5a). The level of activation of p65 increased from 135.7 ± 9.3 to 367.6 ± 98.9 arbitrary units when comparing the data of control cells and Gal-4-treated cells. By using three different inhibitors of this signaling cascade, mRNA levels of IL1B and MMP13 were found to be lowered in their presence (Fig. 5b). To reveal the impact of such reprogramming beyond monolayer (2D) cell culture, we tested Gal-4 in pellet (3D) cultures.

### OA pellets present signs of degradation induced by Gal-4

**Fig. 6:**
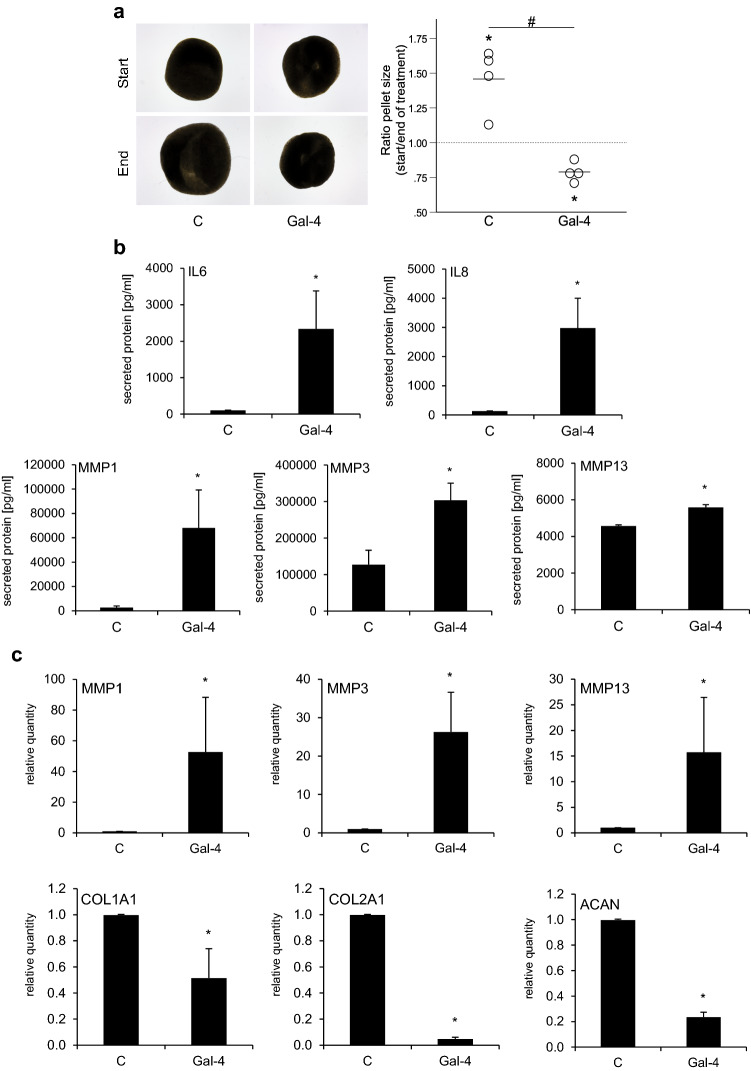
Gal-4 treatment changes pellet size and secretion pattern. **a** Shown are representative images of cultured pellets and the calculated ratio of the pellet size between start (day 21) and end of treatment (day 35). Pellets (*n* = 4 patients) were treated with Gal-4 (12 µg/ml) or were left untreated as control. Each circle represents one patient. Lines indicate the mean values of each group. The dashed line marks the ratio of 1, indicating a hypothetical stable pellet size during treatment. Asterisks indicate the statistically significant difference of a group compared to a value of 1 (“stable size”), whereas number signs indicate significant differences between groups (ANOVA with Tukey post hoc test, *p* < 0.05). **b** OA chondrocyte pellets from four donors were cultured for 3 weeks followed by 2 weeks of treatment with Gal-4 (12 µg/ml). Untreated pellets were used as control. The secreted proteins (MMP-1, MMP-3, MMP-13, IL-6, and IL-8) were measured by a 5-plex human Magnetic Luminex Assay in the supernatants of OA chondrocyte pellets. The asterisk indicates a statistically significant difference between the groups (two-sided paired *t*-test, *p* < 0.05). **c** Bar charts show the relative quantities of mRNA levels of MMP1, MMP3, MMP13, COL1A1, COL2A1, and ACAN (measured using RT-qPCR; 2 technical replicates) in pellets treated with Gal-4 (12 µg/ml) for 7 days (*n* = 5). Significant differences to untreated control pellets (set to 1) are indicated with asterisks (*p* < 0.05, one-sided paired *t*-test)

Gal-4 treatment of 3D OA chondrocyte pellets led to an OA-like occurrence of degradation: Fig. 6a illustrates the appearance of representative pellets (control versus Gal-4 treatment), documenting the reduction in pellet size by Gal-4. Since this effect may result from matrix degradation, we used a 5-plex human Luminex assay to measure the secretion of MMP-1, MMP-3, MMP-13, IL-6, and IL-8 into the supernatant of OA chondrocyte pellets. Here, we found strong upregulation of all matrix degradative proteins, as well as both pro-inflammatory factors (Fig. 6b). By isolating mRNA from pellets treated for one week with Gal-4 and determining the levels of MMP1, MMP3, MMP13, COL1A1, COL2A1, and ACAN transcripts, the presence of MMP1-, MMP3-, and MMP13-specific mRNA was found to be significantly raised, whereas that of both transcripts for collagens and aggrecan was strongly reduced (Fig. 6c).

## Discussion

Faced with the enormous clinical and socioeconomic burden of OA, exploring the nature of effectors within its pathogenesis holds promise to inspire innovative approaches for interfering with disease progression. This study identified Gal-4 as a pathogenic factor in this process in two- and three-dimensional culture systems.

First, the presented immunohistochemical data extend the evidence for an association of Gal-4 presence with cartilage degradation in situ*.* On the cellular level, Gal-4 triggers pro-degradative/-inflammatory responses in an NF-κB-dependent manner, measured by transcriptome analysis, the production of functional disease markers, and the activation of NF-κB signaling. Using 3D (pellet) cultures, we even show a decrease in their size following treatment with Gal-4. These data suggest we should proceed to investigate the following two issues:

- Since relevant post-binding events are known to depend on the context and the nature of the counterreceptor(s) of the galectin, e.g., the α_5_β_1_-integrin for leading to Gal-1-induced p21/p27 accumulation (Fischer et al. 2005) or caspase-8-mediated anoikis (Sanchez-Ruderisch et al. 2011; Amano et al. 2012), counterreceptor identification of cell-surface binding partners for Gal-4 is a means to clarify the initial steps toward the measured increase of specific MMPs and interleukins. In addition to defining the protein part of the binding partner(s), the nature of glycan(s) recruiting the galectin(s) to a lattice formation can then be elucidated. For example, a reduced level of α2,6-sialylation of complex-type *N*-glycans or the presence of core 2 mucin-type *O*-glycans and ganglioside GM1 play crucial roles in the growth regulation of activated T cells and tumor cells by Gal-1 (Kopitz et al. 2001; Cabrera et al. 2006; Valenzuela et al. 2007; Wang et al. 2009; Earl et al. 2010; Amano et al. 2012). Interestingly, OA chondrocytes present diverse types of N-glycans and also core 2 O-glycans in primary culture, with a tendency for an increased degree of N-glycan branching upon further culture (Toegel et al. 2013; Fuehrer et al. 2021). Knowing about contact sites and signaling routes may help to find a new approach to interfere with galectin-dependent disease progression.
- Since (i) high levels of galectins in OA provide docking sites for cognate glycans in situ*,* and (ii) the synthesis of a bifunctional compound presenting lactose, the canonical galectin ligand, and an MMP inhibitor has been reported (Bartoloni et al. 2013), developing high-affinity galectin binders on this platform and applying them in vitro may not only block the harmful galectin binding but also strategically deploy the MMP inhibitor. Our study points to the cooperation of several galectins in OA pathogenesis, adding Gal-4 to the set. Knowing about the complete galectin network is instrumental in designing not just one but a mixture of bifunctional reagents representing the most potent ligands for each galectin, e.g., the aforementioned 3′-*O*-sulfated core 1 mucin-type *O*-glycan disaccharide or the sulfatide headgroup for Gal-4. Oligomers of *N*-acetyllactosamine and its 3′-*O* or 6-*O*-sulfated mono- or bisubstituted derivatives, and these in clustered presentation, will be candidates for Gal-1, Gal-3, and Gal-8 (Hirabayashi et al. 2002; Ideo et al. 2003; Tu et al. 2013; Xiao et al. 2018; Miller et al. 2020).

Collectively, the reported findings disclose a disease-promoting in vitro activity of Gal-4, whose expression is correlated with the extent of OA cartilage degradation in vivo. Thus, Gal-4 joins Gal-1, Gal-3, and Gal-8 in this functional aspect so that the resulting hypothesis of their role in OA pathogenesis as a network merits further study.

## Supplementary Information

Below is the link to the electronic supplementary material.

Supplementary file1 (PDF 147 KB)

Supplementary file2 (XLS 598 KB)

Supplementary file3 (XLSX 41 KB)

Supplementary file4 (XLSX 14 KB)

Supplementary file5 (XLSX 11 KB)

Supplementary file6 (XLSX 10 KB)

Supplementary file7 (XLSX 9 KB)

## Data Availability

The microarray datasets generated and analyzed during the current study are available in the GEO repository (GSE183531). All other data generated or analyzed during this study are included in this published article and its supplementary information files.
