# Supplementary material for: Galectin network in osteoarthritis: galectin-4 programs a pathogenic signature of gene and effector expression in human chondrocytes in vitro
Source: Histochem Cell Biol. 2021 Nov 30;157(2):139–51. doi: 10.1007/s00418-021-02053-1 (PMC8847242; doi:10.1007/s00418-021-02053-1)
Supplement: Supplementary file 1 — Supplementary file1 (PDF 147 KB) [file 418_2021_2053_MOESM1_ESM.pdf]

Supplementary Material and Methods

Methods

Cell viability assay (MTT). Cell viability of chondrocytes was tested after administration of increasing galectin concentrations and CAPE with EASY FOR YOU (EZ4U) assay by Biomedica. In brief, 5000 cells per well were cultivated in a 96-well-plate. At 80% confluency, the treatment was performed in full medium for 24 h at 37 °C. Treatment solutions were removed and cells were incubated with mixed EZ4U substrates for 4 h at 37 °C. Then, the absorbance was measured at 450 nm with FLUOstar optima microplate-reader.

Figure Legends

Figure S1. Cell viability of OA chondrocytes (n=2) treated for 24 h with Gal-4 with ascending doses was measured by metabolic activity.

Figure S2. OA chondrocytes (n=6 patients) were treated for 24 h with 50 µg/ml Gal-4. SDHA was used as reference gene. Asterisks mark significant differences between control and treatments ($p<0.05$, one-sided paired t-test).

Figure S3. Enrichment map of pathways highly regulated by Gal-4, generated by Cytoscape.

Table SI. Computationally detected sites of binding transcription factors in the promoter region and the nine intron sequences of the gene of the human Gal-4 (listed alphabetically). --- please see separate Excel file

Table SII. Listing of sharing of putative binding sites for transcription factors among promoter and intron sequences of the gene of human galectins-1, -3, -4 and -8. The analyzed regions were -2000 to +97 for Gal-1, -2500 for Gal-3, -2441 to +59 for Gal-4 and -2500 for Gal-8 (promoter regions) and the sequences of the introns of the genes

of Gal-1 (three introns), -3 (five introns), -4 (nine introns) and -8 (nine introns). --- please see separate Excel file

Table SIII. Presence of putative binding sites for OA-related transcription factors in promoters and introns of human galectins involved in OA pathogenesis. --- please see separate Excel file

Table SIV. ‘Canonical pathway map’ according to MetaCore analysis resulting from all genes significantly regulated by Gal-4. --- please see separate Excel file

Table SV. ‘Process networks’ according to MetaCore analysis resulting from all genes significantly regulated by Gal-4. --- please see separate Excel file

Table SVI. ‘Diseases’ according to MetaCore analysis resulting from all genes significantly regulated by Gal-4. --- please see separate Excel file

Figure S1

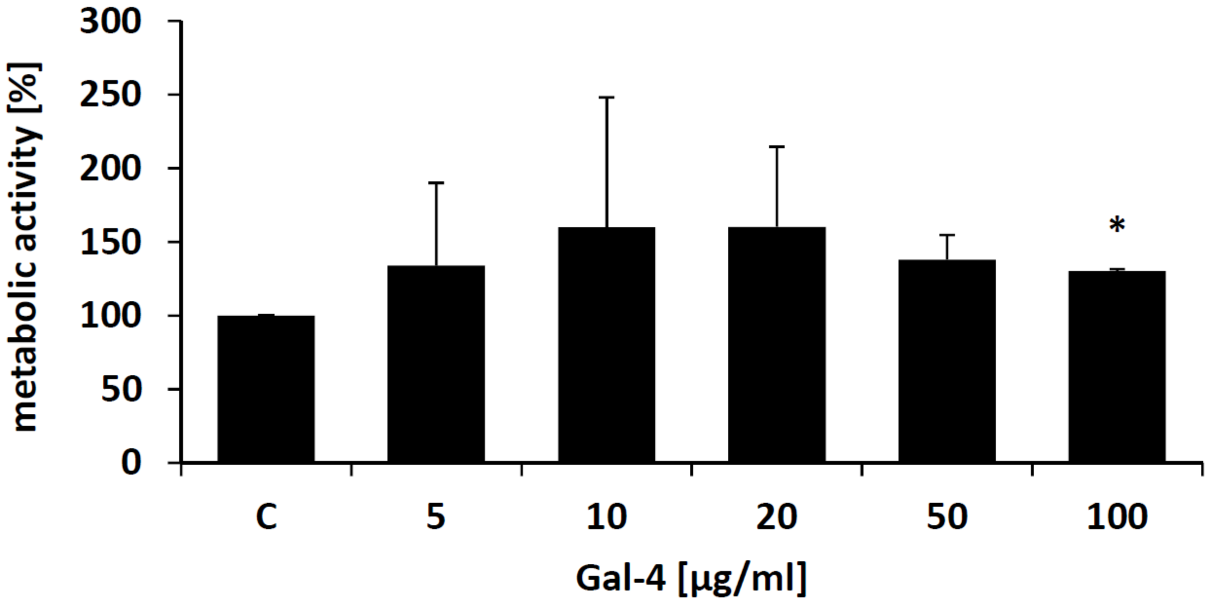

300
250
200
150
100
50
0
metabolic activity [%]
*
C
5
10
20
50
100
Gal-4 [µg/ml]

Figure S2

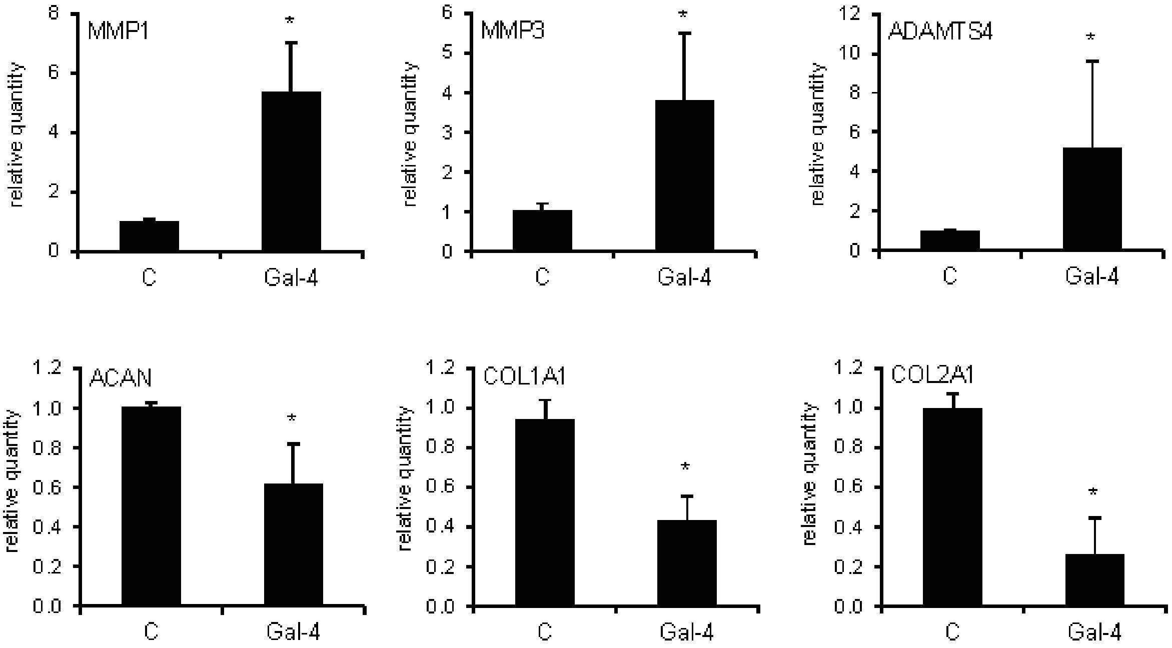
MMP1
MMP3
ADAMTS4
ACAN
COL1A1
COL2A1
relative quantity
C
Gal-4
*

Figure S3

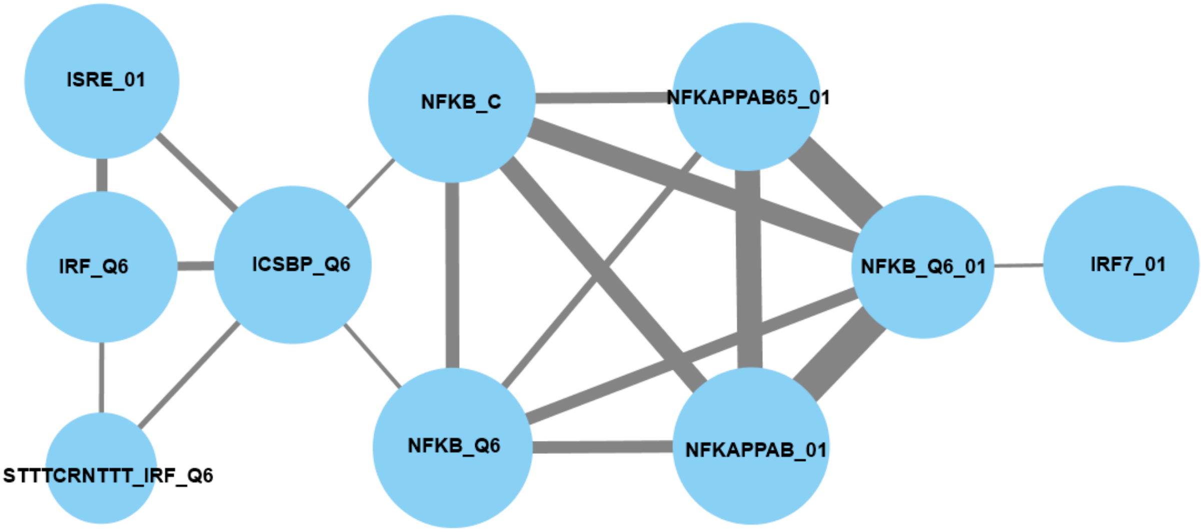
ISRE_01
NFKB_C
NFKAPPAB65_01
IRF_Q6
ICSBP_Q6
NFKB_Q6_01
IRF7_01
NFKB_Q6
NFKAPPAB_01
STTTCRNTTT_IRF_Q6
